# Spatiotemporal prediction of scrub typhus incidence and environmental risk factors in Republic of Korea: a Bayesian hierarchical approach

**DOI:** 10.1186/s40249-026-01481-2

**Published:** 2026-07-23

**Authors:** Youlim Kim, Doheon Kwon, Emmanuel Hasahya, Hu Suk Lee

**Affiliations:** College of Veterinary Medicine, Chungnam National University, 99, Daehak-ro, Yuseong-gu, Daejeon, Republic of Korea

**Keywords:** Disease surveillance, Republic of Korea, Scrub typhus, Environmental risk factors, Bayesian Spatiotemporal modelling

## Abstract

**Background:**

Scrub typhus is a vector-borne infectious disease that is a major public health concern in Republic of Korea. Environmental factors are associated with disease transmission. However, comprehensive predictive models incorporating multiple environmental determinants while accounting for spatial and temporal correlations remain limited. This study aimed to characterize the spatiotemporal patterns of scrub typhus incidence and develop a Bayesian spatiotemporal prediction model incorporating multiple environmental covariates across 229 administrative units in the Republic of Korea from 2015 to 2024.

**Methods:**

We developed a comprehensive analytical framework using national surveillance data from 229 administrative units collected from January 2015 to December 2024. Seasonal-trend decomposition, hotspot analysis, and negative binomial regression were employed to characterize spatiotemporal patterns to inform the Bayesian spatiotemporal modelling framework. Bayesian prediction modelling using an Integrated Nested Laplace Approximation was implemented to analyse environmental covariates, including temperature, relative humidity, normalized difference vegetation index (NDVI), elevation, and cropland ratio, while accounting for spatial clustering and temporal autocorrelation.

**Results:**

Spatiotemporal analyses revealed distinct seasonal dynamics, with November incidence rates 91-fold higher than those in February [incidence rate ratio (IRR): 91.00, 95% confidence interval (*CI*) 66.50–125.00], and significant geographic clustering in southern provinces. The Bayesian model identified significant positive associations between disease risk and temperature [IRR: 1.02 per 1 °C, 95% credible interval (CrI): 1.01–1.04], NDVI (IRR: 1.07 per 0.1-unit, 95% CrI: 1.03–1.11), and elevation (IRR: 1.32 per 100 m, 95% CrI: 1.15–1.51). Relative humidity showed contrasting effects depending on the lag period, with protective effects at a 1-month lag (IRR: 0.96, 95% CrI: 0.93–0.999) but increased risk at a 2-month lag (IRR: 1.16, 95% CrI: 1.12–1.21).

**Conclusions:**

This comprehensive framework successfully captured spatiotemporal disease patterns and provided quantitative risk assessment capabilities for evidence-based public health planning and targeted preventive strategies.

**Supplementary Information:**

The online version contains supplementary material available at 10.1186/s40249-026-01481-2.

## Background

Scrub typhus (ST), also known as tsutsugamushi disease, is an infectious disease caused by the bites of larval trombiculid mites infected with *Orientia tsutsugamushi* [1]. Clinical manifestations include acute fever, chills, headache, and skin eruptions at the mite bite sites. As no vaccination exists, prevention relies on avoiding high-risk areas and using protective measures, such as appropriate clothing and insect repellents, during outdoor activities [2]. ST represents a major public health concern across the Asia–Pacific region, with the highest transmission rates occurring within the “tsutsugamushi triangle,” which extends from the Russian Far East to Pakistan, Australia, and Japan, and includes countries such as Republic of Korea, China, India, Thailand, Indonesia, and the Philippines [3]. Approximately one billion people are at risk of infection, with one million new cases occurring annually in this densely populated endemic region [4].

ST was first documented in Republic of Korea in 1951, when six cases were reported by United Nations military personnel [5]. Following its recognition as an endemic disease, ST has become a significant public health concern, particularly affecting agricultural workers during the harvest season, through transmission mechanisms that remain incompletely understood. In response to its growing impact, the Korea Centers for Disease Control and Prevention designated ST as a Group III notifiable infectious disease in 1994, after which approximately 300 cases have been reported annually [6]. Between 2001 and 2024, Republic of Korea recorded 152,775 cases, demonstrating a clear upward trend. The annual incidence increased from an average of 4667 cases annually during 2005–2010 to 7854 cases during 2011–2020, representing a 68.3% increase [7]. Vector surveillance studies have identified *Leptotrombidium scutellare* as the dominant chigger mite species in Republic of Korea, followed by *Neotrombicula kwangneungensis, N. tamiyai, L. palpale, and L. pallidum* [8, 9]. Among the major chigger mite vectors, the three most abundant species–*L. scutellare, L. pallidum*, and *L. palpale*–exhibit distinct seasonal patterns, with *L. scutellare* occurring predominantly in spring and autumn, *L. pallidum* in autumn, and *L. palpale* in late autumn [8, 9]. These seasonal patterns align with traditional customs in Republic of Korea. The Korean Thanksgiving holiday (Chuseok) typically occurs from late September to early October, coinciding with peak chigger activity. This holiday involves family visits to ancestral burial sites for grave-tending rituals, potentially contributing to increased exposure risk during this period. In addition, these seasonal patterns correspond to temperature-dependent activity, as chigger populations are closely associated with temperature changes [8, 10].

Previous research conducted across endemic regions has established associations between ST and both climatic and environmental factors [11–17]. However, in Republic of Korea, while recent studies have identified key environmental determinants such as temperature, precipitation, and the normalized difference vegetation index (NDVI) [18–22], an integrated analytical framework that incorporates multiple environmental covariates and accounts for spatiotemporal autocorrelation remains underexplored. To address this gap, we implemented the Integrated Nested Laplace Approximation (INLA) framework. Unlike conventional Markov Chain Monte Carlo methods, INLA provides deterministic and computationally efficient approximations to posterior marginal distributions, enabling reliable uncertainty quantification and superior scalability for large spatiotemporal datasets with complex hierarchical random effect structures [23].

Therefore, we aimed to assess seasonality and hotspot patterns, and develop a Bayesian spatiotemporal model of ST incidence in Republic of Korea by integrating multiple environmental covariates and comprehensively accounting for spatial and temporal correlations. To our knowledge, this is the first study to systematically examine the combined effects of multiple environmental determinants in an integrated Bayesian framework for ST prediction in Republic of Korea.

## Methods

### Data sources and acquisition

In this study, monthly surveillance data on ST cases from January 2015 to December 2024 were obtained from the publicly accessible national database maintained by the Korea Disease Control and Prevention Agency (KDCA), which provides open access to national infectious disease statistics without requiring specific permissions or licenses [7]. In Republic of Korea, ST is classified as a Group III notifiable infectious disease under the Infectious Disease Control and Prevention Act, requiring mandatory reporting by all physicians who diagnose patients with ST. Reported cases are reviewed by local health authorities and transferred to the KDCA for final confirmation before being incorporated into the national surveillance database. These data were aggregated into 229 administrative units, including Si, Gun, and Gu, which represent the basic municipal divisions in Republic of Korea, yielding 27,480 spatiotemporal observations based on 229 units over 120 months. Additionally, monthly population data for all 229 administrative units were obtained from the Korean Statistical Information Service [24]. The primary outcome variable was the monthly incidence of ST per 100,000 population in each administrative unit.

The acquisition and spatial aggregation of all environmental covariates were performed using the Google Earth Engine (GEE) cloud computing platform. The GEE is an extensive, publicly available catalogue of satellite imagery and Earth observation data designed for efficient large-scale geospatial analysis [25]. The data processing involved aggregating the monthly values of 229 administrative units. The administrative units varied in spatial extent, with areas ranging from 3.04 km^2^ (Jung-gu, Busan) to 1820 km^2^ (Hongcheon-gun), with a median area of 395.4 km^2^. Based on previous studies, we selected environmental covariates, including mean temperature (°C), mean relative humidity (%), mean NDVI, and total precipitation (mm). Additionally, mean elevation (m), mean urban land ratio (percentage of ‘Urban and Built-up’ pixels), and mean cropland ratio (percentage of combined ‘Croplands’ and ‘Cropland/Natural Vegetation Mosaic’ pixels) were incorporated within each administrative unit boundary (Table 1). Data extraction was conducted using administrative unit polygons (Si, Gun, and Gu) as spatial boundaries, within which pixel-level values were spatially aggregated to polygon-level monthly means using the mean reducer in GEE. For time-varying variables, monthly mean values were computed from all available pixels within each administrative unit for each month, whereas precipitation was aggregated as monthly totals. Static variables (elevation and land cover ratios) were extracted as a single aggregated value per unit and applied across all time points. No missing values were identified for any environmental variable throughout the study period. All environmental variables were resampled to a uniform spatial resolution of 5.5 km to ensure consistency across datasets because the original datasets had varying resolutions. This study was approved by the Institutional Review Board of Chungnam National University (IRB No. 202511-SB-221-01).

**Table 1:** Environmental covariates, source datasets, spatial resolution, and data access information used for spatiotemporal modelling of scrub typhus incidence

Variables	Dataset	Resolution	Resource
Temperature	TerraClimate	4 km	www.climatologylab.org/terraclimate
Precipitation	CHIRPS	5.5 km	www.chc.ucsb.edu/data/chirps
Relative humidity	TerraClimate	4 km	www.climatologylab.org/terraclimate
Elevation	NASA SRTM	30 m	www.jpl.nasa.gov/
NDVI	MODIS	1 km	modis.gsfc.nasa.gov/
Land cover ratio	MODIS	1 km	modis.gsfc.nasa.gov/

### Statistical analysis

#### Spatiotemporal and hotspot analysis

To further decompose the time-series data, we applied seasonal trend decomposition using LOESS (STL) to the nationwide monthly incidence rates per 100,000 population, aggregated across all administrative units, to isolate and examine the secular trend, seasonality, and residual components of the epidemic pattern [26]. To identify the spatial clustering patterns of ST incidence, we conducted hotspot analysis using the Local Getis-Ord Gi* statistic [27]. The analysis was performed on the 10-year mean incidence rates per 100,000 population for each administrative unit, using queen contiguity-based spatial weights with a row-standardised weighting scheme. Hotspots and cold spots were classified based on *z*-score thresholds at 90%, 95%, and 99% confidence intervals (*CI*s), allowing the identification of statistically significant spatial clusters of high and low disease incidence. To quantify temporal variability and identify high-risk months, we pooled the entire 10-year dataset using a mixed-effects negative binomial regression model, with monthly aggregated case counts as the outcome and the log-transformed population (per 100,000) included as an offset term. Month was entered as a categorical fixed effect with February as the reference category, and a random intercept for year was incorporated to account for between-year variability in the pooled data.

#### Environmental data processing and spatiotemporal regression modelling

**Fig. 1:**
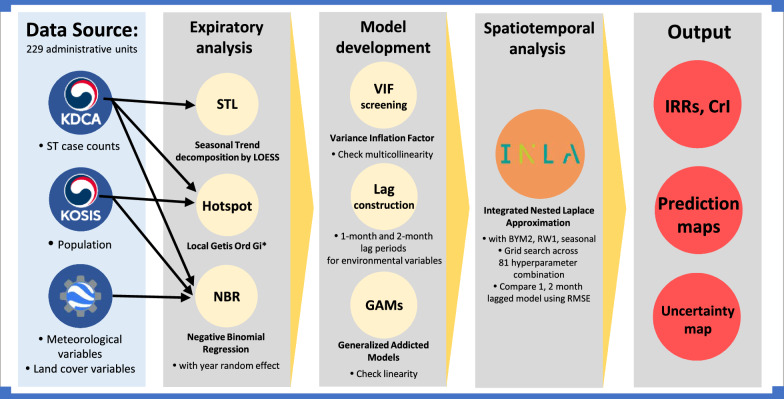
Overview of the data inputs and analytical workflow. *ST* Scrub typhus, *STL* Seasonal-trend decomposition using locally estimated scatterplot smoothing, *NBR* Negative binomial regression, *VIF* Variance inflation factor, *GAM* Generalized additive model, *INLA* Integrated nested Laplace approximation, *IRR* Incidence rate ratio, *CrI* Credible interval

The complete workflow of the data collection, processing, and analytical procedures is shown in Fig. 1. All continuous covariates were standardised by centering them around their mean values and rescaling them as follows: temperature (1 °C increments), relative humidity (10% increments), urban land ratio and cropland ratio (10% increments), precipitation (100 mm increments), elevation (100 m increments), and NDVI (0.1-unit increments). Based on epidemiological evidence of lag effects up to 8 weeks [10, 18], models incorporating 1-month and 2-month lag periods were developed and compared. Prior to model fitting, variance inflation factor (VIF) analysis was conducted for all covariates to assess multicollinearity. Time-varying covariates, including temperature, relative humidity, precipitation and NDVI, were analysed using both 1-month and 2-month lagged terms, whereas the static variables, elevation, urban land ratio and cropland ratio, were included without lagged terms.

To determine the functional form of the environmental covariates, generalised additive models (GAMs) with smoothing splines (k = 6) were fitted for each climatic and environmental variable using Poisson regression. Linearity between environmental covariates and ST incidence was evaluated by examining the GAM-derived smoothing curves. When significant nonlinearity was detected (*P* < 0.05), quadratic terms were incorporated into the model. Overdispersion was confirmed using a likelihood ratio test. Thus, a Bayesian spatiotemporal NBR framework was employed instead of Poisson regression.

We implemented the model using the Integrated Nested Laplace Approximation (INLA)**,** which provides computationally efficient inference for complex hierarchical models compared to traditional Markov Chain Monte Carlo methods [23]. The log of the population was employed as an offset term to account for varying population sizes across administrative units. Two types of random effects were incorporated into the model to address the spatial and temporal dependencies in the surveillance data. Spatial autocorrelation was modelled using the Besag-York-Mollié 2 (BYM2) model structure, separating structured and unstructured spatial variation [28, 29]. Temporal autocorrelation was addressed by applying a first-order random walk (RW1) to the year variable and a seasonal component for the month variable, with a 12-month cycle. The overall model structure is as follows:

$$\log \left( {\mu_{{{\mathrm{it}}}} } \right) = \log \left( {pop_{{{\mathrm{it}}}} } \right) + \alpha + \beta^{\prime}X_{{{\mathrm{it}}}} + b_{{\mathrm{i}}} + \gamma_{{\mathrm{t}}} + \delta_{{\mathrm{s}}}$$

where:

- μᵢₜ = expected case count for area i at time t
- popᵢₜ = population for area i at time t (offset)
- α = intercept
- β′ = regression coefficients for environmental covariates
- Xᵢₜ = environmental covariates
- bᵢ = spatial random effect (BYM2)
- γₜ = temporal structured component (RW1)
- δₛ = seasonal component (12-month cycle)

A comprehensive grid search across 81 hyperparameter combinations was conducted, exploring the spatial mixing parameter (φ: 0.3, 0.5, 0.7), spatial precision parameter (0.20, 0.35, 0.50, 1.5), temporal precision parameter (0.15, 0.20, 0.30), and three different prior specifications for the negative binomial overdispersion parameter (default, loose, tight). Penalized complexity priors were employed for all spatial and temporal hyperparameters. Model performance was evaluated using Watanabe-Akaike Information Criterion (WAIC) and Root Mean Square Error (RMSE). To assess prediction performance, spatial maps comparing the observed and predicted incidence rates were generated for all administrative units using 10-year averaged rates per 100,000 population. Prediction uncertainty was quantified using the relative width of 95% credible intervals (CrIs) and visualised using uncertainty maps. All statistical analyses were performed using R statistical software (version 4.3.1; R Foundation for Statistical Computing, Vienna, Austria) with the following packages: stats, lme4, spdep, R-INLA, and ggplot2.

## Results

### Spatiotemporal patterns and hotspot analysis

**Fig. 2:**
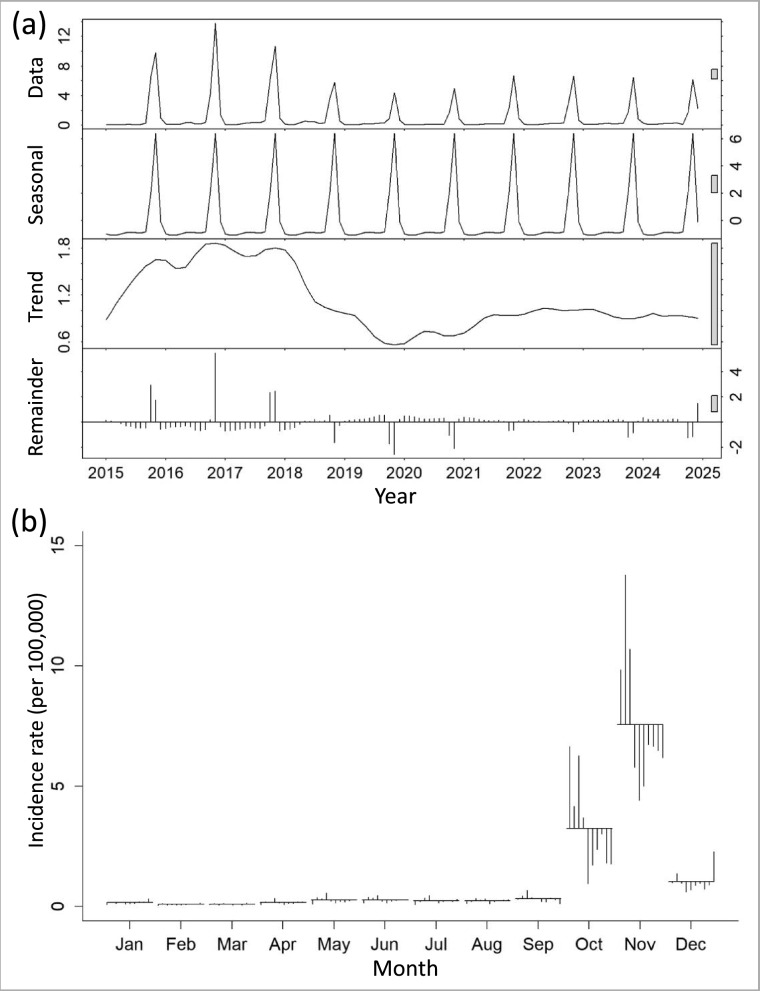
Seasonal trend decomposition using LOESS (STL) decomposition of monthly scrub typhus incidence rates (per 100,000) in Republic of Korea from 2015 to 2024. **a** STL decomposition showing data, seasonal, trend, and remainder components over the study period. **b** Monthly distribution of scrub typhus incidence rates across all study years. STL, seasonal-trend decomposition using LOESS. STL Seasonal-trend decomposition using locally estimated scatterplot smoothing

The STL decomposition of the nationwide monthly ST incidence rates from January 2015 to December 2024 revealed distinct temporal components (Fig. 2). The long-term trend component demonstrated an increase from 2015 to 2018, began declining from late 2018, reached its lowest point between late 2019 and early 2020, started to rise again thereafter, and subsequently stabilised. The seasonal component exhibited a highly consistent annual pattern, with the incidence peaking prominently in autumn across all study years. The cases were heavily concentrated in the autumn months, with minimal disease activity observed from January to August. The incidence began to escalate in September, reaching peak rates per 100,000 population in November before returning to baseline levels during winter.

**Fig. 3:**
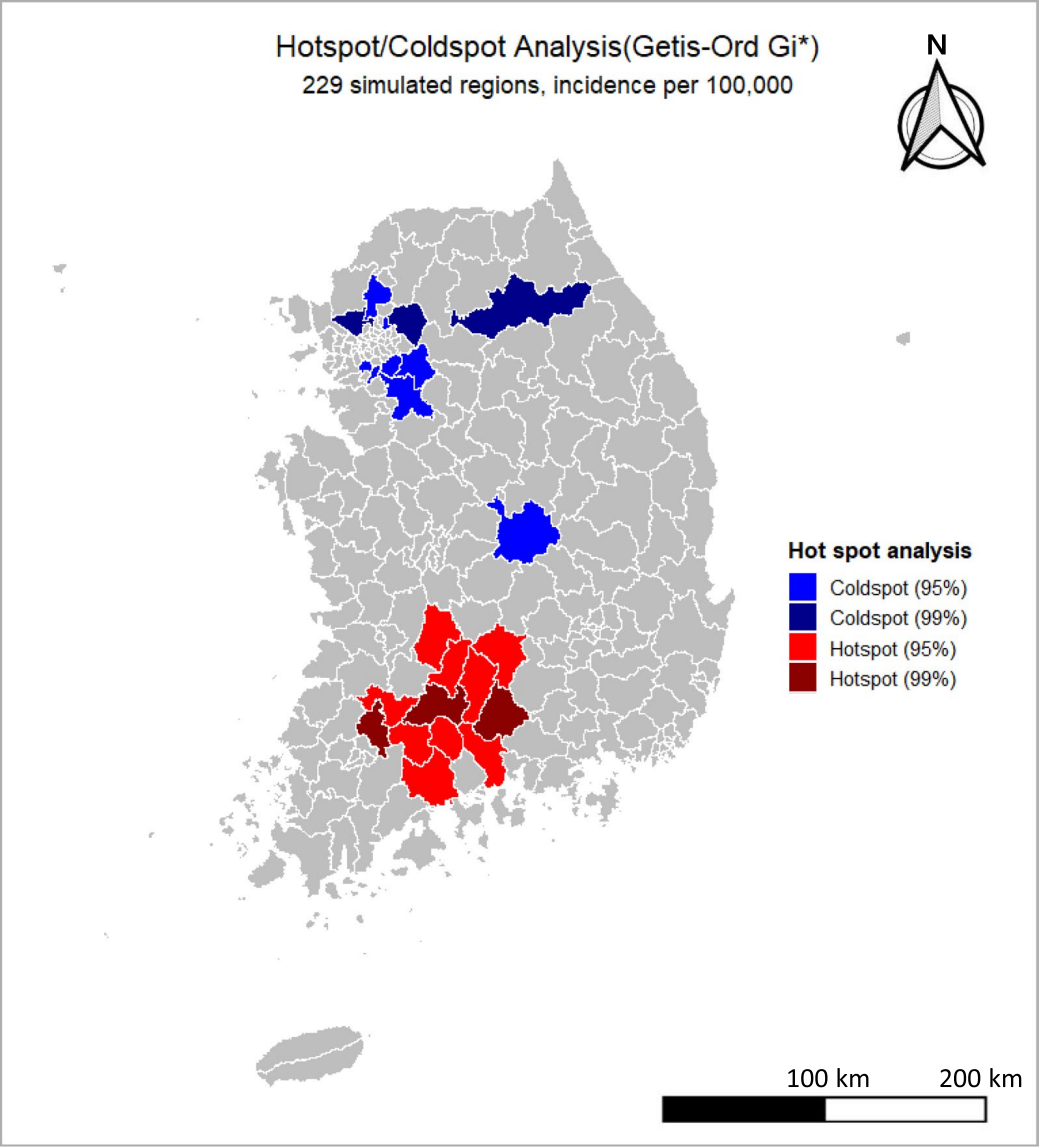
Getis-Ord-Gi* Hotspot analysis of scrub typhus incidence (per 100,000) across Republic of Korea administrative units. *CI* confidence interval, Gi* local Getis-Ord statistic

Hotspot analysis using the Local Getis-Ord Gi* statistic revealed distinct spatial clustering patterns of ST incidence across the 229 administrative units (Fig. 3). High-incidence hotspots (per 100,000) were identified in the southern regions, with significant hotspots (90–99% *CI*) concentrated in areas corresponding to Jeolla and Gyeongsang provinces. Low-incidence cold spots were located in the central and northern regions, including areas around the Seoul metropolitan area and Gangwon Province, with cold spots identified at 90–99% *CI*. The analysis demonstrated clear geographic disparities in disease burden, with southern administrative units showing significantly higher clustering of ST incidences than the northern regions (Fig. 3).

NBR analysis using pooled 10-year data, with February as the reference month, further confirmed the significant seasonal variations in ST incidence (Table 2). Compared with February, all months except March exhibited significantly elevated risk. The risk increases were most pronounced in autumn. June, July, August, and September demonstrated moderate risk elevations, with incidence rate ratios (IRRs) ranging from 2.74 to 3.80. The maximum incidence was observed in November (IRR: 91.00, 95% *CI* 66.50–125.00) and October (IRR: 39.00, 95% *CI* 28.40–53.60), corresponding to 91-fold and 39-fold higher incidence rates, respectively, compared with February. December maintained a significantly elevated risk (IRR: 12.50, 95% *CI* 9.11–17.10) after which the incidence rates returned to baseline during the winter and early spring months.

**Table 2:** Monthly relative risks of ST incidence (IRRs with 95% *CI*)

Month	IRRs	95% *CI*
January	1.86	1.35–2.56
February		reference
March	1.09	0.78–1.51
April	1.88	1.37–2.60
May	2.96	2.15–4.07
June	3.19	2.23–4.38
July	2.79	2.03–3.84
August	2.74	1.99–3.77
September	3.80	2.77–5.23
October	39.00	28.40–53.60
November	91.00	66.50–125.00
December	12.50	9.11–17.10

*IRR* incidence rate ratio, *CI* confidence interval, *ST* scrub typhus

### Spatiotemporal regression modelling

**Fig. 4:**
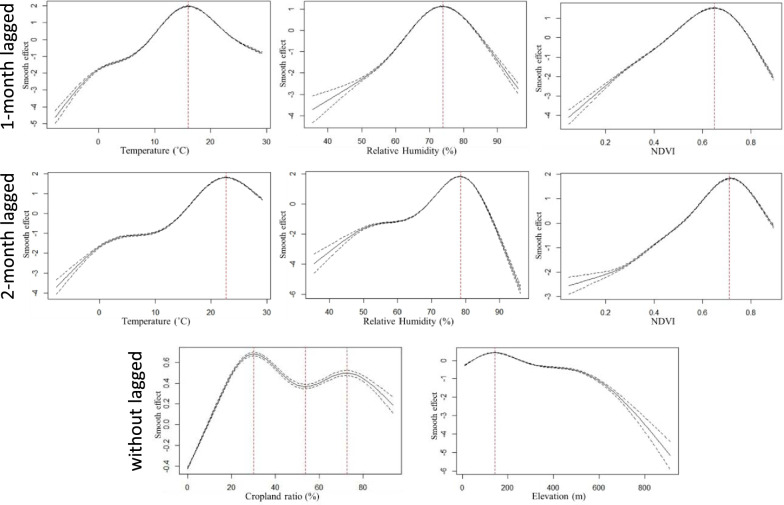
GAM smoothing curves with 95% confidence intervals showing the relationships between environmental variables and scrub typhus incidence at 1-month and 2-month lags. *GAM* generalized additive model, *NDVI* normalized difference vegetation index, *CI* confidence interval

High multicollinearity was identified between precipitation and temperature (VIF > 5), and between urban and cropland ratios (VIF > 5) (Table S1). Although precipitation and urban land ratio may be epidemiologically relevant predictors of ST incidence, they were excluded from the final model to avoid parameter instability and ensure reliable estimation of the remaining covariates. Following their exclusion, all remaining environmental variables exhibited acceptable VIF scores (≤ 5) (Table S2). GAM analysis using smoothing splines revealed nonlinear associations for all remaining environmental variables in both lag models, with effective degrees of freedom values exceeding two for all covariates (Fig. 4, Table S3). Based on these findings, quadratic terms were incorporated for all covariates in the Bayesian spatiotemporal model.

The 1-month lag model demonstrated superior performance with a loose theta prior, spatial mixing parameter (φ) of 0.3, spatial precision parameter of 1.5, and temporal precision parameter of 0.3. The best-fitting combination for the 2-month lag model included a tight theta prior, φ of 0.7, spatial precision parameter of 1.50, and temporal precision parameter of 0.3.

The 1-month lag model revealed the following environmental associations. Temperature showed a positive association with disease risk (IRR: 1.02, 95% CrI: 1.01–1.04), peaking at 16.03 °C. Relative humidity exhibited a protective effect, with peak risk observed at 73.92% (IRR: 0.96, 95% CrI: 0.93–0.999). NDVI was positively associated with disease risk (IRR: 1.07, 95% CrI: 1.03–1.11; peak at 0.65). Elevation also demonstrated a positive association with disease risk (IRR: 1.32, 95% CrI: 1.15–1.51; peak at 142.45 m).

The 2-month lag model presented different patterns than the 1-month model. Temperature maintained a positive association (IRR: 1.03, 95% CrI: 1.02–1.05; peaking at 22.72 °C). In contrast to the protective effect observed in the 1-month model, relative humidity showed a positive association with disease risk, reaching its peak at 78.50% (IRR: 1.16, 95% CrI: 1.12–1.21). NDVI continued to show a positive association (IRR: 1.08, 95% CrI: 1.04–1.12; peak at 0.71), and elevation maintained a positive association (IRR: 1.29, 95% CrI: 1.13–1.48; peak at 142.45 m). The cropland ratio remained non–significant in both lag models. Across both lag models, all significant covariates–temperature, relative humidity, NDVI, and elevation–exhibited inverted U-shaped relationships with ST incidence. Model performance metrics supported the 1-month lag model. Specifically, the 1-month model demonstrated a lower WAIC (62010.39 vs. 62533.26), indicating superior model fit. The RMSE values also favoured the 1-month model (7.08 vs 7.33), suggesting enhanced predictive accuracy for the observed ST incidence rates across administrative units (Table 3).

**Table 3:** Comparison of environmental covariate effects and model performance between 1-month and 2-month lag models

Variables, IRRs (95% CrI)	Lag 1-month model	Lag 2-month model
Temperature	1.02 (1.01–1.04)	1.03 (1.02–1.05)
Relative Humidity	0.96 (0.92–0.99)	1.16 (1.12–1.21)
NDVI	1.07 (1.03–1.12)	1.08 (1.04–1.12)
Elevation	1.32 (1.15–1.51)	1.29 (1.13–1.48)
Cropland ratio	1.05 (0.98–1.11)	1.04 (0.98–1.11)
Model fit metrics		
WAIC	62,010.39	62,533.26
RMSE	7.08	7.33

*IRR* incidence rate ratio, *CrI* credible interval, *NDVI* normalized difference vegetation index, *WAIC* Watanabe-Akaike information criterion, *RMSE* root mean square error, IRRs and 95% credible intervals (CrIs) are presented for linear terms.

Quadratic terms were incorporated to account for nonlinear associations identified in GAM analyses. Units of increase: temperature, per 1 °C; relative humidity, per 10%; NDVI, per 0.1 unit; elevation, per 100 m; cropland ratio, per 10%

**Fig. 5:**
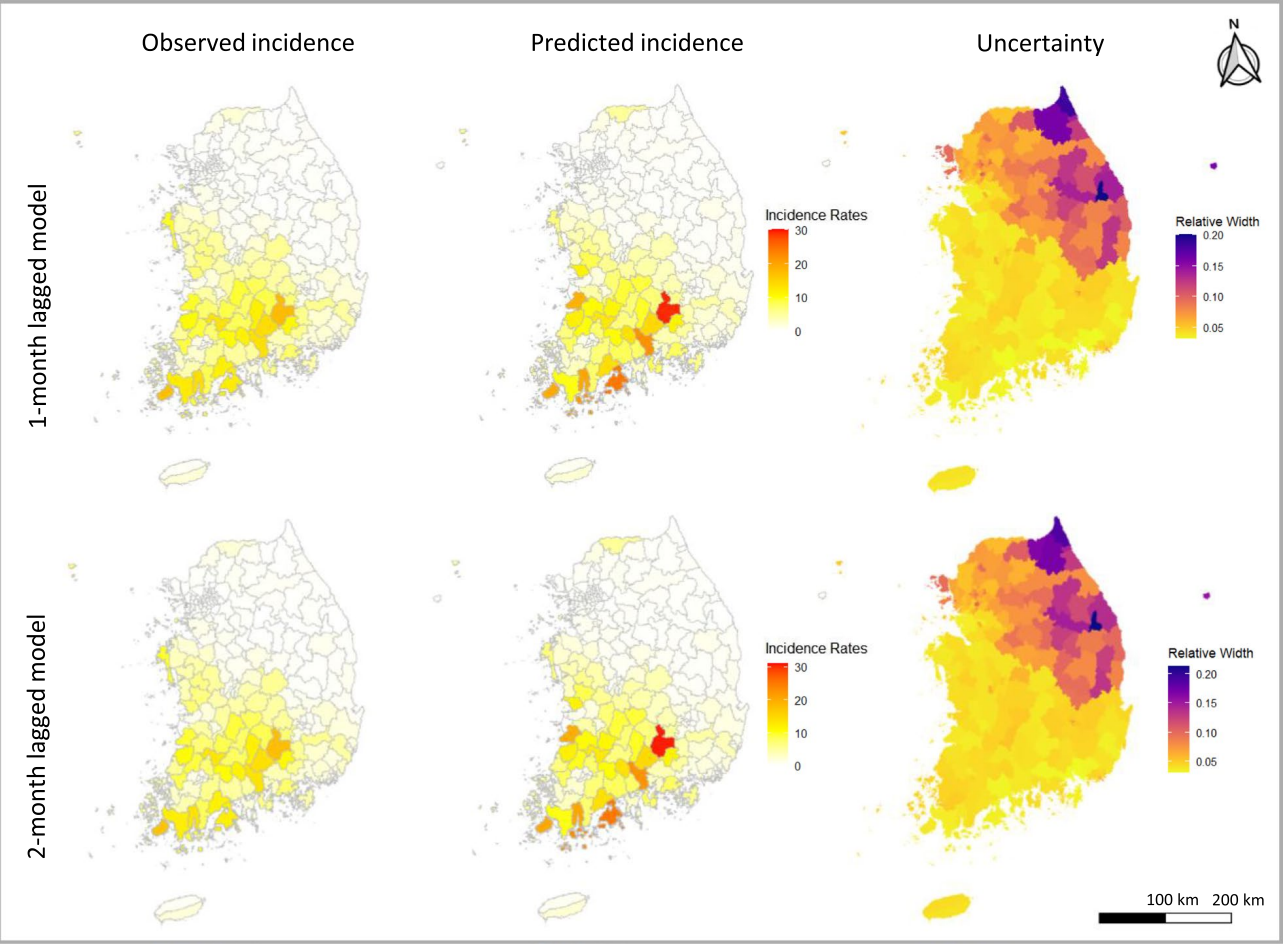
Spatial comparison of observed and predicted scrub typhus incidence rates and associated uncertainty (per 100,000) during the study period of 2015–2024. *CrI* credible interval

The predicted incidence map showed spatial patterns similar to those of the observed data, with both maps showing elevated incidence rates in the southern regions and lower rates in the northern and central areas (Fig. 5). The uncertainty map revealed spatial variation in the prediction confidence (Fig. 5), with higher uncertainty observed in the eastern mountainous regions and some areas with high incidence values, whereas lower uncertainty was evident in areas with moderate incidence rates.

## Discussion

This study developed an integrated Bayesian spatiotemporal prediction model for ST incidence in Republic of Korea, incorporating multiple environmental covariates and accounting for nonlinear relationships through quadratic terms across 229 administrative units over a 10-year period. Although prior research in Republic of Korea has explored various associations between ST and climatic factors, no study has systematically examined the combined effects of environmental determinants in an integrated analytical framework. Recent studies have primarily focused on limited environmental variables, with investigations examining temperature, precipitation, and vegetation measures as primary predictors of disease incidence [18, 19, 21]. Notably, elevation and land-cover ratios have not been evaluated alongside meteorological factors in comprehensive ST models in Republic of Korea. This study addressed this knowledge gap by incorporating a broad spectrum of meteorological and environmental covariates, including temperature, humidity, vegetation index, elevation, and land-use characteristics, into a single modelling framework. Moreover, this analysis extended beyond simple linear associations by implementing quadratic polynomial regression terms to comprehensively capture the complex nonlinear ecological relationships between environmental factors and infection risk.

While previous research established valuable foundations using traditional Bayesian approaches [21], our study introduced several methodological advances to enhance analytical capabilities. Earlier studies employed hierarchical Bayesian models with basic spatial autocorrelation structures [21, 22], whereas our analysis used the INLA framework. We implemented the BYM2 structure combined with RW1 and seasonal components to achieve more efficient and comprehensive spatiotemporal modelling. The BYM2 spatial structure was selected based on hotspot analysis results that revealed significant geographic clustering of ST incidence rates. The temporal autocorrelation components, RW1 and seasonal, were incorporated following STL analysis and negative binomial regression results that demonstrated clear seasonal patterns and temporal trends in the data. Grid search optimization across 81 hyperparameter combinations identified the optimal model specifications, ensuring robust parameter selection and improved reliability of the environmental effect estimates. Additionally, the extended 10-year surveillance period addresses the critical need for long-term forecasting models under varying environmental conditions. Our approach leveraged the cloud-computing capabilities of GEE to extract environmental data directly at the administrative polygon level for all 229 municipal units, ensuring spatial precision aligned with the actual surveillance boundaries. Although the unified 5.5 km resolution resulted in one administrative unit (Jung-gu, Busan: 3.04 km^2^) falling below the extraction resolution, the remaining 228 units maintained adequate spatial resolution for reliable environmental exposure assessment. This framework has significant public health implications, as the identified lag associations between environmental conditions and ST incidence may provide a scientific foundation for forecasting public health responses and support targeted preventive interventions in high-risk areas.

Our analysis revealed significant positive associations between ST incidence and temperature, NDVI, and elevation, which were consistently observed across both 1-month and 2-month lag models. The positive association with temperature aligns with previous findings indicating that meteorological conditions are linked to chigger mite ecology through effects on life cycle processes, oviposition, and activity patterns [10, 30]. Studies in Republic of Korea have reported positive correlations between temperature and ST occurrence, with temperature being a critical factor for chigger mite ecology because of its effects on egg hatching rates and developmental speed within suitable temperature ranges [18, 31]. Similarly, NDVI and elevation exhibited positive associations with ST incidence, highlighting their interconnected relationship with disease transmission. Increased vegetation coverage provides favourable habitat conditions and food resources for rodent populations and chigger mite communities, and studies have shown that vegetation expansion leads to an increased abundance of these key ecological components in the transmission cycle [13, 21]. Rodents, which serve as primary vertebrate hosts, preferentially inhabit areas with dense vegetation that provide food and protection from predators [32]. The positive association with elevation is closely related to vegetation dynamics, as altitude is associated with vegetation density, which may in turn be linked to disease occurrence patterns [15]. Higher altitudes are associated with an increased ST incidence owing to denser vegetation growth and elevated relative humidity, where vegetation remains less disturbed by human activities [33]. Although precipitation has been consistently identified as an important environmental predictor in previous ST studies, it was excluded from our analysis because of its high multicollinearity with other climatic variables (VIF > 5), which ensured model stability and reliable parameter estimation.

Relative humidity exhibited contrasting associations depending on the lag period, suggesting complex mechanisms involving both human behavioural and ecological dynamics. The negative association in the 1-month lag model aligned with previous findings, where ST incidence declined with increasing humidity, contrary to the expectation of higher mite abundance in wet environments [14, 34]. This phenomenon may reflect human behavioural responses to environmental conditions, as high-humidity periods often coincide with reduced outdoor activities, leading to decreased exposure to chiggers and a lower infection risk. In contrast, the positive association observed in the 2-month lag model reflects delayed ecological effects on vector populations. This temporal shift corresponds to chigger life cycle dynamics, with temperature and humidity serving as critical factors in chigger development, as complete development from egg to infective larvae requires approximately 6–8 weeks [10]. The 2-month lag period suggests that favourable humidity conditions improve chigger survival and reproduction, with the epidemiological impact manifesting approximately 2 months later.

Our study revealed a notably higher prediction uncertainty in Gangwon Province than in other regions (Fig. 5). This elevated uncertainty likely reflects distinct ecological patterns in the northern mountainous regions compared to the southern endemic areas, where most ST cases occur. The chigger species composition in northern regions, specifically Gangwon Province, showed marked differences from that in major endemic regions, such as the southern provinces. Among the eight known ST vector species in Republic of Korea, Gangwon Province exhibited lower vector species diversity and a substantially reduced abundance of key species, such as *L. palpale*, compared with the southern endemic regions. Moreover, *L. tectum* was predominantly found in the northern regions, whereas *N. kwangneungensis* was highly abundant in Gangwon Province [8, 9]. These regional variations in vector species distribution may contribute to different transmission dynamics that are not fully captured by the model parameters optimised for southern endemic patterns. Additionally, the limited healthcare infrastructure in Gangwon Province, with substantially fewer hospital beds, with a mean of 17,630 beds compared to major southern endemic provinces such as Jeollanam-do with 41,036 beds and Gyeongsangnam-do with 62,447 beds based on 2015–2023 averages [35], may contribute to the underdiagnosis and underreporting of ST cases, resulting in higher prediction uncertainty in the northern regions.

This study had some limitations. First, ST cases may be subject to underreporting, particularly in rural and vegetated areas, where the disease is most prevalent. Patients may not seek medical care due to limited healthcare access, mild or nonspecific symptoms, or misdiagnosis. According to the World Health Organization, ST is a substantially underdiagnosed and underreported cause of hospitalised febrile illnesses in endemic regions [36]. Second, our analysis was conducted at the administrative unit level (Si, Gun, and Gu), which lacks individual-level information, including socioeconomic factors, specific biting sites, and personal risk behaviours that may be associated with infection risk. This ecological study design is subject to ecological fallacy, limiting the ability to make valid inferences about individual-level relationships using aggregate data [37]. Third, potential exposure misclassification may have arisen from the resampling and spatial aggregation of environmental covariates to a uniform 5.5 km resolution, which may not fully capture fine-scale environmental heterogeneity within administrative units. Fourth, the absence of comprehensive chigger mite distribution data limited our ability to directly validate the relationship between the predicted environmental suitability and actual vector abundance across different regions. This gap between environmental modelling and vector surveillance constrains model validation and accuracy in vector-borne disease studies. Despite these limitations, the findings of this study may serve as a basis for forecasting public health planning and inform evidence-based allocation of preventive resources for ST prevention in Republic of Korea.

Future research should prioritize the integration of comprehensive entomological surveillance data with environmental modelling to improve prediction accuracy. Additionally, incorporating individual-level risk factors and socioeconomic determinants would provide a more nuanced understanding of transmission dynamics and enhance the applicability of these models to regional disease control programs.

## Conclusions

This study established the first comprehensive Bayesian spatiotemporal framework for ST prediction in Republic of Korea and demonstrated that environmental factors showed significant associations with disease transmission patterns. Our analysis revealed pronounced seasonal dynamics, with incidence rates in November being 91-fold higher than the baseline levels, alongside distinct geographic clustering in the southern provinces. The modelling framework identified temperature, NDVI, and elevation as consistent positive risk factors for disease transmission, while relative humidity exhibited protective effects at a 1-month lag but increased disease risk at a 2-month lag. These findings provide critical insights into the environmental drivers of ST transmission and offer quantitative risk assessment capabilities for evidence-based public health planning. This analytical framework represents a significant methodological advancement in vector-borne disease modelling in Republic of Korea and provides a foundation for improved surveillance and prevention strategies.

## Supplementary Information

Supplementary Material 1.Table S1. Variance inflation factorvalues for all candidate environmental covariates prior to variable selection. Table S2. Variance inflation factorvalues for environmental covariates retained in the final model following exclusion of multicollinear variables. Table S3. Effective degrees of freedomand significance of smoothing terms from generalized additive modelanalyses for retained environmental covariates

## Data Availability

The data that support the findings of this study are openly available in the Korea Disease Control and Prevention Agency (KDCA) Infectious Disease Portal at https://www.kdca.go.kr/eng/, reference number [7]; the population data are available in the Korean Statistical Information Service at https://kosis.kr/eng/, reference number [24]; the environmental data are available in the public domain through the Google Earth Engine platform at https://earthengine.google.com/, reference number [25].

## Abbreviations

ST: Scrub typhus
NDVI: Normalized difference vegetation index
INLA: Integrated nested Laplace approximation
KDCA: Korea Disease Control and Prevention Agency
STL: Seasonal-trend decomposition using LOESS
LOESS: Locally estimated scatterplot smoothing
*CI*: Confidence interval
IRR: Incidence rate ratio
VIF: Variance inflation factor
GAM: Generalized additive model
NBR: Negative binomial regression
BYM2: Besag–York–Mollié 2
RW1: First-order random walk
WAIC: Watanabe-Akaike information criterion
RMSE: Root mean square error
CrI: Credible interval
GEE: Google Earth Engine
