# Supplementary material for: Spatiotemporal prediction of scrub typhus incidence and environmental risk factors in Republic of Korea: a Bayesian hierarchical approach
Source: Infect Dis Poverty. 2026 Jul 23;15:82. doi: 10.1186/s40249-026-01481-2 (PMC13393755; doi:10.1186/s40249-026-01481-2)
Supplement: Supplementary file 1 — Supplementary Material 1.Table S1. Variance inflation factorvalues for all candidate environmental covariates prior to variable selection. Table S2. Variance inflation factorvalues for environmental covariates retained in the final model following exclusion of multicollinear variables. Table S3. Effective degrees of freedomand significance of smoothing terms from generalized additive modelanalyses for retained environmental covariates [file 40249_2026_1481_MOESM1_ESM.docx]

**Spatiotemporal prediction of scrub typhus incidence and environmental risk factors in Republic of Korea: a Bayesian hierarchical approach**

Youlim Kim, Doheon Kwon, Emmanuel Hasahya, Hu Suk Lee

**Table of contents**

**Table S1.** Variance inflation factor (VIF) values for all candidate environmental covariates prior to variable selection

**Table S2.** Variance inflation factor (VIF) values for environmental covariates retained in the final model following exclusion of multicollinear variables

**Table S3.** Effective degrees of freedom (EDF) and significance of smoothing terms from generalized additive model (GAM) analyses for retained environmental covariates

**Table S1.** Variance inflation factor (VIF) values for all candidate environmental covariates prior to variable selection

| **variables** | **1-month lagged** | **2-month lagged** |
| --- | --- | --- |
| Temperature | 6.02 | 6.07 |
| Relative Humidity | 2.26 | 2.27 |
| Precipitation | 6.72 | 6.72 |
| NDVI | 5.20 | 5.22 |
| Elevation | 5.03 | 5.03 |
| Cropland ratio | 4.82 | 4.83 |
| urban ratio | 7.39 | 7.40 |
| Temperature (Quadratic) | 1.31 | 1.32 |
| relative humidity (Quadratic) | 1.26 | 1.26 |
| Precipitation (Quadratic) | 6.72 | 3.35 |
| NDVI (Quadratic) | 1.13 | 1.13 |
| Elevation (Quadratic) | 2.58 | 2.58 |
| Cropland ratio (Quadratic) | 3.54 | 3.54 |
| Urban ratio (Quadratic) | 4.20 | 4.20 |

Note: Variables with VIF > 5 were considered to indicate unacceptable multicollinearity and were excluded from the final model.

| **variables** | **1-month lagged** | **2-month lagged** |
| --- | --- | --- |
| Temperature | 3.93 | 3.95 |
| Relative Humidity | 1.88 | 1.89 |
| NDVI | 4.21 | 4.22 |
| Elevation | 4.22 | 4.22 |
| Cropland ratio | 3.48 | 3.48 |
| Temperature (Quadratic) | 1.27 | 1.27 |
| Relative humidity (Quadratic) | 1.14 | 1.14 |
| NDVI (Quadratic) | 1.10 | 1.10 |
| Elevation (Quadratic) | 2.51 | 2.51 |
| Cropland ratio (Quadratic) | 3.42 | 3.42 |

**Table S2.** Variance inflation factor (VIF) values for environmental covariates retained in the final model following exclusion of multicollinear variables

Note: All remaining variables exhibited acceptable VIF scores (≤ 5) following exclusion of multicollinear variables.

**Table S3.** Effective degrees of freedom (EDF) and significance of smoothing terms from generalized additive model (GAM) analyses for retained environmental covariates

| **variables** | | **EDF** | ***P*** |
| --- | --- | --- | --- |
| 1-month lagged | Temperature | 4.96 | <.001 |
|  | Relative humidity | 4.81 | <.001 |
|  | NDVI | 4.89 | <.001 |
| 2-month lagged | Temperature | 4.93 | <.001 |
|  | Relative humidity | 4.93 | <.001 |
|  | NDVI | 4.92 | <.001 |
| Without lagged | Elevation | 4.89 | <.001 |
|  | Cropland ratio | 4.95 | <.001 |
